# Single-port laparoscopic sacrospinous ligament suspension *via* the natural vaginal cavity (SvNOTES) for pelvic prolapse: The first feasibility study

**DOI:** 10.3389/fsurg.2022.911553

**Published:** 2022-07-18

**Authors:** Yuanyuan Lyu, Huafeng Ding, Jin Ding, Yonghong Luo, Xiaoming Guan, Guantai Ni

**Affiliations:** ^1^ Department of Obstetrics and Gynecology, The First Affiliated Hospital of Wannan Medical College, Wuhu, China; ^2^ Department of Obstetrics and Gynecology, The Second Affiliated Hospital of Harbin Medical University, Harbin, China; ^3^ Department of Obstetrics and Gynecology, Baylor College of Medicine, Houston, TX, United States

**Keywords:** pelvic floor dysfunction (PFD), sacrospinal ligament fixation, surgery, single-port laparoscopic, natural vaginal cavity

## Abstract

**Objective:**

This study aims to investigate the feasibility and short-term efficacy of single-port laparoscopic-assisted transvaginal natural cavity endoscopic sacrospinous ligament suspensions (SvNOTES).

**Methods:**

A total of 30 patients diagnosed with anterior or/and middle pelvic organ prolapse Stages III and IV underwent natural vaginal cavity (SvNOTES), and 30 patients who underwent conventional sacrospinous ligament (SSLF) were used as a control group. The operation time, blood loss, postoperative POP-Q score, length of hospital stay, and complications were compared between the two groups.

**Results:**

The operation time for SvNOTE was (60 ± 13) min, which was longer than (30 ± 15) min for SSLF (*P* = 0.04). However, the bleeding amount in SvNOTE was 29.44 ± 2.56, significantly lower than that in the SSLF group (80 ± 10; *P *= 0.02), and the postoperative hospital stay in the SvNOTE group was (4 ± 2) days, longer than (3 ± 1) days in SSLF (*P *= 0.02). However, there were no intraoperative complications in the SvNOTE group, whereas one ureteral injury occurred in the SSLF group; in addition, the postoperative POP-Q score was significantly better in the SvNOTE group than that in the SSLF group with increasing time (*P* < 0.001).

**Conclusion:**

Compared with SSLF, single-port laparoscopic sacrospinous ligament suspension *via* the natural vaginal cavity is visualized, greatly improving the success rate of sacrospinous ligament fixation, with less blood loss and fewer complications, arguably a safer and minimally invasive surgical approach.

## Introduction

Pelvic floor dysfunction (PFD) is a series of functional disorders caused by the development of defects, weakness, degradation, damage, and dysfunction of the pelvic floor support structure, which leads to pelvic organ displacement and comprises the social activity of patients (1, 2). As the aging started to increase in China, the incidence rate of PFD has increased significantly. As a result, it has become a common disease that seriously affects the quality of life of middle-aged and older women.

In recent decades, novel minimally invasive surgery technologies have been developed. Due to the lower level of postoperative pain, short hospitalization time, rapid recovery, and a good cosmetic effect, natural orifice transluminal endoscopic surgery (NOTES), a novel minimally invasive surgical method, has gained preference, and a large number of reports have been published on this technique (3). In this surgical method, the abdominal cavity is entered through natural orifices, such as the mouth, urethra, and anus, without any abdominal incisions being made (4, 5). Compared with laparoscopic surgery, NOTES has the advantages of being minimally invasive, eliminating the need for any abdominal incisions and reducing postoperative abdominal pain, incision infection, hernia formation, and adhesion (6).

Most NOTES operations can be performed *via* the vagina or oral cavity. Since the vagina can be easily decontaminated and provides direct access, safety, and easy closure, transvaginal NOTES has been widely used for cholecystectomy, nephrectomy, and various gynecological diseases (7–9). In addition, NOTES has been commonly used in several surgical procedures not only by gynecologists (10) but also by general surgeons and has been proven safe and easy to close (11, 12).

The management of pelvic organ prolapse includes sacrocolpopexy, high uterosacral ligament suspension, and sacral spinal ligament fixation (SSLF). Publications have demonstrated that conventional or robotic NOTES sacrocolpopexy and high uterosacral ligament suspension are feasible and safe. However, there have been only a few reports published on the *sacrospinous ligament suspension* treatment of pelvic organ prolapse *via* NOTES.

Therefore, the purpose of this study was to compare the feasibility, safety, efficacy, and cosmetic effect of transvaginal endoscopy and conventional treatment for pelvic organ prolapse.

## Methods

### General Information

A total of 60 patients with Grade III or above anterior or middle pelvic prolapse admitted to the Department of Obstetrics and Gynecology of the First Affiliated Hospital of Wannan Medical University from January 2020 to 2021 were selected. All patients were postmenopausal women who presented to our obstetrics and gynecology department for symptomatic vaginal vault prolapse or uterine prolapse. Patients who had a diagnosis of gynecologic tumor or malignancy requiring open surgery or laparoscopy, who had an untreated vaginal infection, or who could not be followed up for 2 years at the beginning of the trial were excluded. Patients included in the trial underwent detailed urogynecological and medical history investigations before surgery. We assessed patients’ urological, bowel symptoms, and sexual life by means of relevant questionnaires using the Pelvic Floor Impact Questionnaire-Short Form 7 (PFIQ-7) and the Pelvic Organ Prolapse/Urinary Incontinence Sexual Questionnaire (PISQ-12). The main questions included symptoms related to prolapse, such as sensation of the vaginal bulge, pelvic pressure, difficulties in voiding the bladder or bowel, urinary incontinence, coital frequency, dyspareunia, and possible effect of prolapse and surgery postoperatively on sexual function (13).

Patients were randomly assigned to one of two groups. The first group received fixation of the vaginal vault with the sacrospinous ligament, and the second group received single-port laparoscopic sacrospinous ligament suspension *via* the natural vaginal cavity. A computer-generated randomization list was developed by a statistician. Preoperative randomization was performed by an independent nurse who removed the card from one patient, blinded. The nurse removed a card from an opaque envelope.

### Preoperative preparation

After admission, cervical cytology was performed to rule out cervical disease and ultrasound was performed to rule out uterine and bilateral adnexal disease (e.g., uterine fibroids, adnexal masses, etc.). A potassium permanganate sitz bath was given 3 days before surgery. One day before surgery, an oral magnesium sulfate retardant was given to prevent infection induced by leakage of intestinal contents due to intestinal injury during surgery.

## Surgical methods

### Vaginal sacrospinous ligament fixation

All patients underwent combined spinal and epidural anesthesia in the lithotomy position with the patient's legs kept buckled to prevent abduction. Then, 50 ml of normal saline was injected into the posterior vaginal mucosa and the rectal space to create a water cushion to separate the pelvic tissue from the right sciatic spine and the right sacral spine ligament. The right sacral spine ligament was exposed. The cervical tissue attached to the sacral ligament was suspended at the midpoint of the right sacral spine ligament using a no. 7 silk suture, and the vaginal mucosa was sutured using a 2/0 absorbable vicryl suture.

### Transvaginal natural orifice transluminal endoscopic surgery

**Figure 1:**
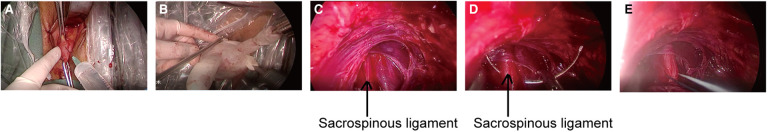
(**A**) Make a single incision after separating the gap. (**B**) Placement of homemade single-hole port. (**C**) Find and isolate the sacrospinous ligament. (**D,E**) Suture of the sacrospinous ligament.

The position of the posterior vaginal fornix was determined. Next, 50 ml of saline was injected into the rectovaginal space using the needle in the posterior vaginal fornix to form a water dissection and cushion. An electric knife was used to cut the whole layer out of the rear wall of the vagina, which was about 3 cm long, and a single-hole port (need to put port company's name here) was inserted and secured. CO_2_ was insufflated at 10 mmHg to maintain sufficient pneumovagina. An ultrasonic blade was used to separate each layer of the vaginal wall to approach the sacral spine ligament. During the operation, nearby blood vessels and nerves were avoided, and the sacral spine ligament was found to be 2 cm long, according to the sciatic spine. Then, a 1-0 absorbable suture needle was placed under direct visualization through the ipsilateral sacral spine ligament and fixed on the cervical main sacral ligament or vaginal stump (Figure 1).

If the patient had vaginal anterior and posterior wall bulging, vaginal anterior and posterior wall repair was performed accordingly. If a preoperative diagnosis of stress urinary incontinence was provided, intraoperative transobturator tension-free vaginal tape was applied. The same experienced surgeon performed all operations.

## Statistical methods

SPSS 21.0 software was used for all statistical analyses. Measurement data were expressed as mean ± standard deviation (*χ* ± S), and variance analysis was used to compare data between groups. The enumeration data were expressed as percentages, and the *χ*^2^ test was performed on data between groups. A *P* < 0.05 was considered statistically significant.

## Results

### Basic situation of patients

As shown in Table 1, there were no significant differences in age, body mass index, parity, and uterine prolapse between the two groups (*P* > 0.05). The POP-Q at each indicator point (Aa, Ba, C, Ap, and Bp, as shown in Table 1) was recorded.

**Table 1:** Characteristics of the study patients (*n* = 101).^a^

Characteristics	SSLF (*n* = 30)	SvNOTES (*n* = 30)	*P*-value
Age (years)	68 ± 5.2	68 ± 4.8	0.08
BMI	25 ± 4.3	29 ± 5.1	0.04
Delivery times	3 ± 1	2.5 ± 1.5	0.52
Preoperative POP-Q scores			
Aa	0.23 ± 1.0	0.31 ± 1.2	0.13
Ba	0.3 ± 1.1	0.4 ± 1.3	0.21
Ap	−0.6 ± 1.2	−0.4 ± 1.5	0.14
Bp	−0.5 ± 2.0	−0.4 ± 1.8	0.19
C	2.0 ± 1.7	2.6 ± 1.8	0.02
TVL	4.9 ± 0.3	6.1 ± 1.9	0.25
POP stage before the operation			
II	15	9	0.22
III	10	15	0.17
IV	5	6	0.30

^a^ *Values are given as mean ± SD, number, number (percentage), or median (range), unless indicated otherwise*.

### Intraoperative situation

In the SvNOTES group, the blood pressure of one patient decreased to 50/20 mmHg and blood oxygen saturation decreased to 30% after the formation of elevated air pressure during the operation. The procedure was immediately stopped, and conventional surgery was performed instead. In the SSLF group, one patient had a ureteral injury. There were no bladder and rectum injuries or complications such as large blood vessel and nerve injury in either of the two groups. The operation time in the SSLF group was 30 ± 15 min, while that in the SvNOTES group was 60 ± 13 min (*P* = 0.04), with a significant statistical difference between the two groups. Intraoperative blood loss in the SSLF group was 80 ± 10 ml, while that in the SvNOTES group was 20 ± 14 ml (*P* = 0.02), with statistical significance (Table 2).

**Table 2:** Perioperative outcomes by the surgical group.^a^

Outcome	SSLF (*n* = 30)	SvNOTES (*n* = 30)	*P*-value
Decrease in oxygen saturation	0	1	–
Conversion to surgery	0	1	–
Blood loss (ml)	80 ± 10	20 ± 14	**0.02**
Operative time (minutes)	30 ± 15	60 ± 13	**0.04**
Postoperative stay (days)	3 ± 1	4 ± 2	**0.02**
Injury	1	0	–
Postoperative fever	1	0	**0.004**
Pain	3	7	**0.003**
Delayed hemorrhage	0	0	–
Infection	0	0	–
Abscessus	0	0	–

^a^ *Values are given as mean ± SD or number (percentage) unless indicated otherwise*.

*Bold value indicated p value was meaningful.*

### Short-term postoperative complications

The residual urine level was 110 ± 12 ml in the SSLF group and 90 ± 10 ml (*P* = 0.01) in the SvNOTES group. Patients with a residual urine level of more than 100 ml continued to retain catheterization, and electrical stimulation physiotherapy was performed. One week later, the catheter was removed again. The indwelling catheterization time was 7 ± 2 days in the SSLF group and 4 ± 1 days in the SvNOTES group (*P* = 0.003). There were no significant differences (Table 3) in postoperative normal activity time and postoperative body temperature stability time.

**Table 3:** Postoperative complications.^a^

	SSLF (*n* = 30)	SvNOTES (*n* = 30)	*P-*value
Stress urinary incontinence (SUI)			
Before surgery	15	18	0.45
After surgery	–	–	–
Improved	10	16	**0.02**
Persistent or worsened	2	0	–
Indwelling time of the catheter (days)	7 ± 2	4 ± 1	**0.003**
Residual urine (ml)	110 ± 12	90 ± 10	**0.01**
Relapse of prolapse	4	1	

^a^ *Values are given as number (percentage) or number unless indicated otherwise*.

*Bold value indicated p value was meaningful.*

### Postoperative objective observation indexes and subjective symptom scores

After 24 months of follow-up, the Aa, Ba, C, Ba, and Bp were recorded (Table 4). The pelvic dysfunction scale (PFDI-20) was used to evaluate pelvic function. We found that the difference in postoperative PFDI-20 scores between the two groups was statistically significant (*P* < 0.05). The pelvic organ prolapse disorder scale (POPDI-6), defecation dysfunction scale (CRADI-8), Pelvic Floor Impact Questionnaire-7 (PFIQ-7), and urinary dysfunction scale (UDI-6) were also collected. In addition, the sexual function scale (FSFI) was used to evaluate the quality of sexual life (the higher the score, the higher the quality of sexual energy). In this study, the pelvic function of the SvNOTE group was significantly better than that of the SSLF group (Table 5).

**Table 4:** Postoperative objective observation indexes.^a^

POP-Q	SSLF (*n* = 30)	SvNOTES (*n* = 30)	*P*-value
6 months			
Aa	−2.6 ± 0.4	−3.0 ± 0.2	0.63
Ba	−2.5 ± 0.3	−2.8 ± 0.3	0.53
Ap	– 2.5 ± 0.2	−2.9 ± 0.3	0.36
Bp	−2.7 ± 0.1	−2.7 ± 0.2	0.28
C	−7.0 ± 0.5	−8.0 ± 0.5	<**0.001**
TVL	7.2 ± 0.7	8.1 ± 0.4	<**0.001**
12 months			
Aa	−2.3 ± 0.3	−2.6 ± 0.5	0.32
Ba	−2.2 ± 0.3	−2.5 ± 0.3	0.23
Ap	–2.2 ± 0.2	−2.4 ± 0.4	0.55
Bp	−2.5 ± 0.2	−2.4 ± 0.1	0.44
C	−6.9 ± 0.3	−7.7 ± 0.2	<**0.001**
TVL	6.9 ± 0.5	7.8 ± 0.1	<**0.001**
24 months			
Aa	−2.2 ± 0.8	−2.3 ± 0.3	0.06
Ba	−2.0 ± 0.6	−2.2 ± 0.3	0.13
Ap	−2.0 ± 0.7	−2.2 ± 0.3	0.71
Bp	−2.1 ± 0.5	−2.2 ± 0.8	0.54
C	−6.7 ± 0.4	−7.5 ± 0.4	<**0**.**001**
TVL	6.7 ± 0.5	7.4 ± 0.2	<**0**.**001**

^a^ *Values are given as mean ± SD unless indicated otherwise*.

*Bold value indicated p value was meaningful.*

**Table 5:** Subjective symptom score at the 2-year follow-up.^a^

Outcome	SSLF (*n* = 30)	SvNOTES (*n* = 30)	*P*-value
PFDI-20 score			
Preoperative	16.21 ± 3.92	16.41 ± 4.31	0.31
2-year follow-up	13.83 ± 0.35	11.02 ± 0.31	0.02
*P-*value	**0.006**	**<0.001**	–
PFIQ-7 score			
Preoperative	193.61 ± 46.09	194.68 ± 45.21	0.59
2-year follow-up	90.6 ± 10.5	70.7 ± 10.2	0.01
*P-*value	**<0.001**	**<0.001**	–
POPDI-6 score			
Preoperative	35.8 ± 9.8	37.8 ± 10.5	0.56
2-year follow-up	9.9 ± 1.8	6.8 ± 1.7	0.02
*P-*value	**<0.001**	**<0.001**	–
UDI-6 score			
Preoperative	29.4 ± 2.5	30.9 ± 2.8	0.66
2-year follow-up	9.8 ± 1.5	6.5 ± 1.5	0.01
*P-*value	**<0.001**	**<0.001**	–
CRADI			
Preoperative	25.51 ± 3.1	25.89 ± 3.4	0.08
2-year follow-up	12.2 ± 2.1	8.1 ± 2.7	0.03
*P-*value	**<0.001**	**<0.001**	–
FSFI score			
Preoperative	5 ± 2	4 ± 3	0.45
2-year follow-up	20 ± 3	30 ± 2	0.03
*P-*value	**0.03**	**0.02**	–

^a^ *Values are given as mean ± SD unless indicated otherwise*.

*Bold value indicated p value was meaningful*.

### Postoperative complications

The recurrence of pelvic prolapse refers to Degree II or above prolapse after 6 weeks of operation. In the SSLF group, the recurrence rate was 13.3% (4/30) (there were three cases with Stage I and one case with Stage II pelvic organ prolapse). In the SvNOTES group, only one case of recurrence of pelvic organ prolapse was reported. After investigation, both operations were confirmed as recurrence of anterior pelvic prolapse (Table 3). The symptoms of the 10 patients with urinary incontinence in the SSLF group improved significantly, while that of two cases did not improve. V-NOTE combined with urinary incontinence was ultimately improved, with no new cases of urinary incontinence (Table 3).

## Discussion

In clinical practice, transvaginal surgery was difficult for some obese patients or patients with vaginal stenosis, transvaginal NOTES is particularly suitable for those patients. The first clinical transvaginal NOTES transvaginal endoscopic cholecystectomy was performed by Zorrón et al. (14) at the University Hospital of Teresopolis, Brazil. Thereafter, Bessler et al. (15) performed the same at Columbia University Medical Center, New York, USA, and Marescaux et al. (16) performed the same at University Louis Pasteur, Paris, France, using similar procedures. Currently, the use of NOTES for gynecologic surgery, including transvaginal hydrolaparoscopy, transgastric adnexectomy, and tubal ligation (6, 17), has steadily advanced. However, there have very few reports on pelvic floor disorders, especially the sacrospinous ligament. To our knowledge, this is the first case series in which the sacrospinous ligament was fixed using SvNOTES. The successful completion of the procedure demonstrates the feasibility of a transnatural cavity with single-port laparoscopy to effectively improve the symptoms of pelvic organ prolapse in women.

Compared with conventional transvaginal surgery, the surgical ﬁeld of SvNOTES can be clearly visualized using an endoscopic camera; any pathology beyond the reach of the operator can be easily managed with the assistance of laparoscopic instruments (18, 19). More importantly, it has the advantage of the absence of abdominal scarring (20).

As we all know, SSLF is a classic surgical procedure used for the treatment of pelvic prolapse (21), but the location of the sacral spine ligament is profound and its anatomy is complex (22, 23). This involves many nerves, internal pudendal arteries, sacral plexus vessels, and ureter, and the rectum is located near the sacral spinous ligament (24). Therefore, blind dissection and suturing may cause vascular nerve ureteral injury (25). In addition, the SSLF procedure is more difficult in patients who are infertile, obese, or have vaginal stenosis; one case in which the suture needle was broken and lost during the operation, resulting in reoperation due to postoperative pain, has been previously reported (26). In our research group, it was found that compared with SSLF, abdominal sacrospinal ligament suspension has the disadvantages of a longer operation time, significant trauma, slower recovery, and higher cost. Therefore, we designed SvNOTE based on operations that are currently performed. In our study, we found that this surgical method leads to a lower recurrence rate and fewer complications. Due to better visualization of vital anatomy during SvNOTES, the incidence of ureteral injury decreased significantly (no ureteral injuries were reported in this study). In addition, the location of the intraoperative suture needle was constantly monitored, which significantly decreased the probability of suture needle loss.

In addition, the operation space available during SSLS is narrow, and the whole operation is wholly sutured or punctured based on the operator's experience. This may result in differences or errors in the anchoring position, which will lead to an increased postoperative recurrence rate. After SvNOTE surgery was implanted into the single-port laparoscopic PORT through the natural cavity, the sacrospinal ligament was exposed under direct visualization. Therefore, the blood vessels, nerves, and urethra surrounding the sacrospinal ligament were safely dissected. In addition, the intraoperative pneumoperitoneum can push the rectum away from the surgical field, which further helps in avoiding essential organs and blood vessels. In addition, when a small amount of bleeding is found during the operation, we can stop the bleeding in time, resulting in minimal intraoperative bleeding. Finally, since we sutured the exposed sacrospinal ligament to the top of the vagina, we could accurately anchor it in a specific position and strengthen it for the future. Our study demonstrated that the intraoperative bleeding of patients was significantly less in the SvNOTE group than that in the control group. The postoperative recurrence rate decreased significantly, and the postoperative recovery time of patients was not longer than that of the control group. Therefore, our surgical technique is safer and produces a lower recurrence rate than SSLS.

However, after port implantation, air pressure was used to expand the field of vision. The increase in air pressure may lead to the possibility of gas embolism. During our operation, the blood pressure of one of the patients dropped sharply after inflation. After immediate blood gas analysis, gas embolism was considered. After the data were analyzed, it was found that the intraoperative air pressure was slightly higher (13 mmHg). Therefore, the air pressure was controlled at 8–10 mmHg during the rest of the SvNOTE operations (27), and the blood oxygen saturation was closely monitored during the procedure by actively cooperating with anesthetists to ensure the safety of the operation. Since this is a single-hole process, the surgeon should not only be familiar with the pelvic floor structure but should also master the single-hole technology to avoid the chopstick effect. As this was a single-hole operation that required the bilateral sacral spine ligament to be clearly exposed, the operation time was longer than that of the conventional group. In addition, intraoperative attention should not be paid to arbitrary electrocoagulation or cutting, and membrane anatomy should be preserved as much as possible. Otherwise, adjacent organs may be damaged due to an unclear level of vision caused by bleeding.

SvNOTES are especially beneﬁcial for patients considered as relative contraindications in conventional transvaginal surgery due to the restricted downward traction of pelvic organs for surgical manipulation and hemostasis (28). SvNOTES allow for scar lessness and exert a lesser degree of triangulation loss and instrument crowding due to vaginal elasticity. A surgeon who wishes to perform SvNOTES should be conﬁdent in PFD. Experience in PFD will certainly lower the learning curve and shorten the operative time.

However, our procedure has several drawbacks similar to those of conventional sacrospinous ligament suspension surgery, such as the vaginal axial bias to the side of suspension and posteriority, which increases the risk of anterior pelvic prolapse. However, our procedure can be performed through a natural channel using a single-hole laparoscope, which makes it minimally invasive or even noninvasive. In addition, we can precisely expose the sacrospinous ligament under direct vision and perform sacrospinous ligament fixation. This makes the anchoring position highly accurate, which greatly reduces the postoperative recurrence rate. Furthermore, intraoperative pneumoperitoneum pressure is used to push open the rectum, and the use of the laparoscope results in important blood vessels and nerves being avoided, which significantly reduces the incidence of surgical complications. In addition, since a mesh was not used during surgery, there is no risk of mesh erosion.

In conclusion, single-port laparoscopic sacrospinous ligament suspension *via* the natural vaginal cavity for pelvic prolapse is an original adding something new to the literature in the field of minimally invasive surgery for pelvic organ prolapse surgery. Our surgical approach improved surgical precision, significantly reduced the recurrence rate, and led to a significant decrease in intraoperative complications. The limitations of our study are the small sample size and the high operator requirements (proficiency in operating single-port laparoscopic surgery and great familiarity with pelvic floor anatomical findings). However, with the comparison group, we can validate the safety and efficacy of this procedure.

## Data Availability

The original contributions presented in the study are included in the article/Supplementary Material, further inquiries can be directed to the corresponding author/s.
